# Dose Constraints in Carbon-Ion Radiation Therapy to Minimize the Risk of Pectoral Myositis

**DOI:** 10.1016/j.ijpt.2025.100746

**Published:** 2025-03-27

**Authors:** Noriyuki Okonogi, Kumiko Karasawa, Kazutoshi Murata, Takuma Sayama, Ikumi Furuichi, Hitoshi Ishikawa

**Affiliations:** 1 Department of Radiation Oncology, Juntendo University Graduate School of Medicine, Tokyo 113-8421, Japan; 2 QST Hospital, National Institutes for Quantum Science and Technology, Chiba 263-8555, Japan; 3 Department of Radiation Oncology, Kawakita General Hospital, Tokyo 166-0001, Japan; 4 Accelerator Engineering Corporation, Chiba 263-8555, Japan

**Keywords:** Pectoral myositis, Dose constraint, Linear energy transfer, Carbon-ion radiation therapy

## Abstract

**Purpose:**

Carbon-ion radiation therapy (C-ion RT) is an emerging nonsurgical treatment for early stage breast cancer, offering biological advantages such as high linear energy transfer (LET) and precise dose distribution. However, the risk of radiation-induced toxicity, particularly pectoral myositis, remains unclear. This study evaluates the relationship between RBE-weighted dose, LET, and pectoral myositis in patients receiving C-ion RT.

**Patients and Methods:**

Fourteen patients with cT0-1N0M0 breast cancer were treated with C-ion RT (46-50 Gy [RBE]) in the prone position. Magnetic resonance imaging was performed pretreatment and at 3-month intervals post treatment to assess pectoral myositis. RBE-weighted dose-volume histograms and LET distributions were analyzed. Statistical comparisons between patients with and without pectoral myositis were conducted using chi-square and t-tests.

**Results:**

Four of 14 patients (29%) developed pectoral myositis, all within 3 months of treatment. Higher RBE-weighted doses in small volumes of pectoralis major muscle were significantly associated with myositis (D_2 cm^3^_ >10 Gy [RBE], *P* = .014). The D_0.1__cm^3^_ to the pectoralis major muscle in patients without pectoral myositis was consistently below 33 Gy (RBE). However, LET distributions showed no significant correlation with myositis development.

**Conclusion:**

This study is the first to evaluate pectoral myositis after C-ion RT for breast cancer. The findings suggest that high RBE-weighted doses in small muscle volumes increase the risk of myositis, while LET is not a significant factor. Establishing dose constraints for the pectoralis major muscle is crucial to minimize radiation-induced toxicity. Further studies with larger cohorts are needed to validate these results.

## Highlights

• We analyzed if pectoral myositis after carbon-ion radiation therapy is dose-dependent.
• Higher doses in small volumes of the muscle were associated with pectoral myositis.
• A high rate of pectoral myositis was observed when D_2 cm^3^_ exceeded 10 Gy (RBE).
• No correlation was observed between pectoral myositis and the linear energy transfer.

## Introduction

Breast-conserving surgery followed by postoperative radiation therapy (RT) is a standard treatment for early stage breast cancer. However, for patients who are not surgical candidates due to age, comorbidities, or personal preference, nonsurgical ablative treatments, such as radiofrequency ablation, focused ultrasound, and cryotherapy, are emerging as viable alternatives.^1^, ^2^ Recently, research on the use of carbon-ion radiation therapy (C-ion RT) as a treatment option for early stage breast cancer is advancing.^3^, ^4^

Unlike conventional X-ray therapy, C-ion RT offers unique biological and physical advantages. Carbon-ion beams have a higher linear energy transfer (LET), resulting in densely clustered DNA damage that is more difficult for cancer cells to repair.^5^ This increases the biological effectiveness of C-ion RT, making it 2 to 3 times more potent than X-ray therapy.^6^ The physical properties of C-ion beams, including a sharp dose fall-off beyond the Bragg peak, enable highly conformal dose distribution, reducing radiation exposure to surrounding normal tissues.^7^ These unique characteristics make C-ion RT a promising alternative for breast cancer treatment, especially in patients unable to undergo surgery.^3^, ^4^

As described above, C-ion beams have distinct biological effects, necessitating new dose constraints to prevent radiation-induced toxicity. The pectoralis muscle is a critical organ at risk during breast RT. Several case reports have indicated the risk of pectoralis myositis and necrosis following stereotactic body RT.^8^, ^9^ In some cases, it can also be painful and negatively impact the patient's quality of life.^8^, ^9^ While dose constraints for the lungs and ribs have been established in photon RT, information on dose constraints for pectoral myositis remains limited. Moreover, there have been no reported cases of pectoralis myositis associated with C-ion RT. Herein, we report the need for dose constraints on the pectoralis muscle during radical C-ion RT for early stage breast cancer treatment.

## Patients and methods

### IRB approval and registration of research

The original study design was approved by the Institutional Review Board (ID: L20-001) and conducted in accordance with the principles outlined in the Declaration of Helsinki. Before the start of the study, the study protocol was registered and published in the University Hospital Medical Information Network, Clinical Trials Registry (UMIN000041032).^10^ Patients were recruited at our hospital, and provided written informed consent prior to enrolling in the present study. Although the original study is still ongoing, new findings from it were assessed in this study. Specifically, we analyzed whether pectoral myositis induced by C-ion RT is dose-dependent or related to LET. This study was approved by the institutional protocol committee.

### Patient characteristics

Between December 2020 and October 2023, 15 patients with early stage breast cancer were treated using C-ion RT. Of these, 14 patients who were treated in the prone position were enrolled in the present study. In accordance with the protocol, all patients enrolled were histologically proven cT0-1N0M0 breast cancer.^10^ Median age at enrollment was 57 (range; 50-72) year-old. Of 14 patients enrolled, 11 patients had invasive ductal carcinoma of the breast, and the remaining 3 had ductal carcinoma in situ. All invasive ductal carcinomas were Luminal A-like. The median follow-up period after C-ion RT was 24.2 months (range; 9-43). Patient and tumor characteristics and treatments are shown in Supplementary Table 1.

### Treatment and follow-up

This was a phase I/II study, with phase I consisting of a dose escalation study and phase II consisting of treatment with the recommended dose determined in phase I. All patients received C-ion RT level I: 46 Gy or level II: 50 Gy (relative biological effectiveness; RBE) in a single fraction, followed by hormone therapy based on their breast cancer subtypes. According to the clinical trial protocol, magnetic resonance imaging (MRI) was performed before and 3 months after C-ion RT. Subsequently, MRI was performed every 3 months until a clinically complete tumor response was observed. The patient was examined on the day of the MRI procedure.^10^

### RBE dose calculation and LET distributions

The modified microdosimetric kinetic model was applied for RBE-weighted dose calculations at our institution and is expressed in Gy (RBE) in this study.^11^, ^12^ The data acquisition method for dose-averaged LET (LETd) distributions was similar to that used in our previous studies.^13^, ^14^ Briefly, the RBE-weighted dose distributions based on the modified microdosimetric kinetic model were calculated using the XiO-N treatment planning system (Mitsubishi Electric, Tokyo), and the LETd was calculated from the RBE and physical dose.^15^ Primary carbon ions and secondary and tertiary projectile nuclear fragments were counted in LETd using the Sihver model.^16^ LETd at location **r** was calculated as follows:

$$\overset{®}{L}\left(r\right)=\frac{\sum_{i}\left[n_{i}∙D_{i}(r)∙L_{i}(r)\right]}{\sum_{i}\left[n_{i}∙D_{i}(r)\right]},$$

where *Di*(**r**) denotes the physical dose distribution for beam *i*, *ni* denotes the beam fraction, and *Li*(**r**) denotes the LET distribution for beam *i*. *Li* in the equation is the LETd of the *i*-th beam at location **r**: The LET for this study was defined using the following settings: unrestricted, LET for water, and no-density normalization.^17^

### Data collection

Pectoral myositis was diagnosed upon examination by the physician in charge and based on a combination of MRI findings. In this study, pectoral myositis was defined based on changes in a contrast-enhanced MRI of the irradiated area. The pectoral myositis in this study was graded according to the following criteria: grade 1 was defined as MRI changes without associated symptoms, while grade 2 was defined as MRI changes accompanied by pain. A radiation oncologist contoured the pectoralis major muscle on the treated side. The RBE-weighted dose parameters of the pectoralis muscle were obtained using dose-volume histograms. We also obtained an LETd volume histogram for the pectoralis muscle. The RBE-weighted dose for small volumes was evaluated using D_Xcm^3^_ Gy (RBE), which represents the minimum RBE-weighted dose at which a volume of X cm^3^ was irradiated.

### Statistical analyses

The chi-square test was used to compare the 2 groups. F-tests were used to determine whether the variances in the data were equal. Student's t-tests were performed when data normality was confirmed and equal variances were assumed. Welch’s t-test was used to analyze unequal variance. All test results were considered statistically significant at a 2-sided *P* value < .05. SPSS 27.0 (for Mac) was used for statistical analyses (IBM Corp, Armonk, New York).

## Results

**Figure 1:**
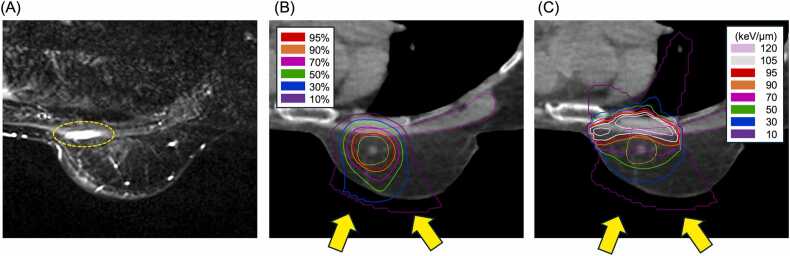
A pectoral myositis and dose distributions of C-ion RT. (A) A representative MRI of pectoral myositis after 3 months of C-ion RT. (B) RBE-weighted dose distribution of C-ion RT on the CT image for the same patient. Isodose lines indicate percentages of the prescribed dose (50 Gy [RBE]). (C) LETd distribution of C-ion RT on the CT image for the same patient. Isodose lines indicate the values of LETd. Pectoral myositis was determined based on changes in a contrast-enhanced MRI of the irradiated area, as indicated by the yellow dotted line (A). The pectoralis major muscle is surrounded by a magenta line. Yellow arrows indicate the direction of the C-ion RT (B) and (C). Abbreviations: C-ion RT, carbon-ion radiation therapy; CT, computed tomography; LETd, dose-averaged linear energy transfer; MRI, magnetic resonance imaging; and RBE, relative biological effectiveness.

Four of the 14 analyzed patients had pectoral myositis; 3 patients had grade 1 myositis, and 1 patient had grade 2 myositis. All myositis was present within 3 months of C-ion RT. Figure 1 shows the MRI images and dose distribution in the case of pectoral myositis. Comparisons between patients with and without pectoral myositis revealed no significant differences in patient characteristics or treatment (Supplementary Table 1).

All RBE-weighted dose-volume histograms and LETd volume histogram data for the 14 patients are shown in Supplementary Figure 1. Focusing on the small volume of the pectoralis major muscle, the RBE-weighted dose histogram showed a trend towards higher doses in patients with pectoral myositis (Supplementary Figure 1A). However, LET volume histograms showed no specific trends (Supplementary Figure 1B). Table 1 compares the histogram parameters of the RBE-weighted dose volume between patients with and without pectoral myositis. When comparing RBE-weighted dose-volume parameters between patients with and without pectoral myositis, patients with pectoral myositis showed a statistically significant difference in D_2__cm^3^_ Gy (RBE) (*P* = .014) and D_0.1__cm^3^_ Gy (RBE) (*P* = .015) (Table 1). Ten (91%) of the 11 cases showed no pectoral myositis when D_2__cm^3^_ was below 10 Gy (RBE). The D_0.1__cm^3^_ to the pectoralis major muscle in patients without pectoral myositis was consistently below 33 Gy (RBE).

**Table 1:** Comparisons of the histogram parameters of RBE-weighted dose-volume between patients with and without pectoral myositis.

Variable	n = 10 (w/o pectoral myositis)	n = 4 (w/ pectoral myositis)	*P*-value
D_5__cm^3^_, average±SD, Gy (RBE)	0.9 ± 1.6	2.9 ± 4.0	.396
D_2__cm^3^_, average±SD, Gy (RBE)	2.5 ± 4.6	13.4 ± 10.3	.014
D_1__cm^3^_, average±SD, Gy (RBE)	3.9 ± 6.9	22.5 ± 16.0	.099
D_0.1__cm^3^_, average±SD, Gy (RBE)	7.6 ± 12.1	33.5 ± 22.5	.015

**Abbreviations:** w/o, without; w/, with; D_Xcm^3^_, the minimal radiation doses for the most irradiated volumes of X cm^3^; SD, standard deviation; and RBE, relative biological effectiveness.

**Figure 2:**
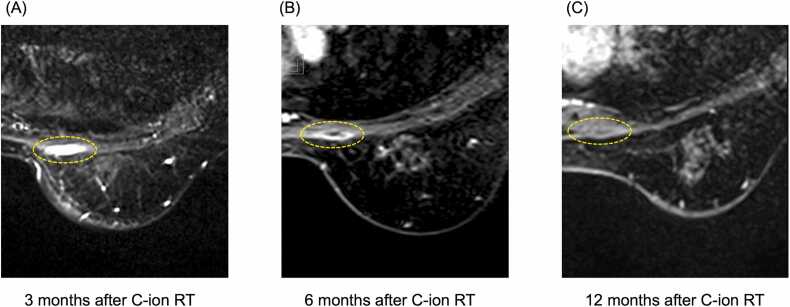
The typical course of pectoral myositis after C-ion RT. Pectoral myositis appeared at 3 months after C-ion RT (A). The condition persisted at 6 months after C-ion RT (B) but resolved by 12 months after C-ion RT (C). Yellow dotted lines indicate the area of pectoral myositis. Abbreviation: C-ion RT, carbon-ion radiation therapy.

The typical course of pectoral myositis is shown in Figure 2. As mentioned above, when pectoral myositis occurs, it typically presents within 3 months of C-ion RT. The condition did not resolve at 6 months after C-ion RT but disappeared by 12 months after C-ion RT. In cases of grade 2 pectoral myositis, which involves pain, the progression of pain generally corresponded with changes observed in MRI images. Specifically, pain peaked at 3 months after C-ion RT and then gradually improved, resolving by 12 months after C-ion RT.

## Discussion

To the best of our knowledge, this is the first study to evaluate pectoral myositis after C-ion RT. The use of C-ion RT has increased significantly in recent years.^18^ Although several dose constraints in C-ion RT have been reported,^19^, ^20^, ^21^ this is the first report on the relationship between pectoral myositis and RBE-weighted dose or LETd values. Given that patients undergoing nonsurgical treatment for breast cancer are a vulnerable population who are medically inoperable, less invasive care is desirable. Ablative treatment of breast cancer with C-ion RT is becoming established.^3^, ^4^ Therefore, this study is significant because it revealed the presence of pectoral myositis caused by C-ion RT.

Our study suggests that high RBE-weighted doses in small volumes of the pectoralis major muscle are associated with postirradiation pectoral myositis. In particular, a high rate of pectoral myositis was observed when D_2 cm^3^_ exceeded 10 Gy (RBE). It should be noted that the dose-impairment relationship was similar to that of serial organs rather than parallel organs. Combining our findings and considering the availability of reports on rib fractures but the absence of reports on pectoral myositis in C-ion RT for lung cancer,^22^ pectoral myositis is more strongly associated with small volumes than with medium-to-large volumes. However, if the endpoints, such as pectoral muscle contracture, change instead of pectoral myositis, the indicators may also change. Therefore, further evaluation of multiple endpoints in a larger study population is required.

It may also be important to note that there was no association between pectoral myositis and LETd values in this study. Recently, RBE-weighted doses considering LETd have been reported to be helpful in proton beam therapy for predicting late adverse reactions in the brain and ribs.^23^, ^24^, ^25^, ^26^ Meanwhile, we reported that LETd values per se were not associated with late rectal or pelvic bone adverse reactions to C-ion RT.^13^, ^14^ The results of this study are consistent with those of our previous study. The true nature of the relationship between LETd and adverse reactions is still unclear, as it involves diverse factors such as the type of particle beam, differences in target organs, and methods of calculating LET, and further research is needed.

This study has some limitations owing to its limited sample size and the inclusion of a single institution. Therefore, further evaluation with a larger number of patients and, if possible, multicenter validation is necessary. In conclusion, we demonstrated that higher RBE-weighted doses in small volumes of the pectoralis major muscle were associated with postirradiation pectoral myositis. In particular, a high rate of pectoral myositis was observed when D_2 cm^3^_ exceeded 10 Gy (RBE). However, no correlation was observed between pectoral myositis and the LETd. Our results highlight the importance of considering the dose constraints on the pectoralis major muscle in patients receiving C-ion RT.

## Ethics

All patient data have been collected under an internal review board approved protocol.

## Funding

This study was supported by a Grant-in-Aid for Scientific Research (23H02869, 23K27560) from the Ministry of Education, Culture, Sports, Science and Technology of Japan.

## Author Contributions

Noriyuki Okonogi: Conceptualization, Methodology, Formal analysis, Investigation, Writing- Original Draft, Visualization, Funding acquisition. Kumiko Karasawa: Conceptualization, Writing- Review & Editing, Supervision. Kazutoshi Murata: Validation, Investigation, Writing- Review & Editing. Takuma Sayama: Data Curation. Ikumi Furuichi: Software, Data Curation. Hitoshi Ishikawa: Supervision, Project administration.

All authors commented on the manuscript and have read and approved the final version.

## Declaration of Conflicts of Interest

Noriyuki Okonogi reports financial support was provided by the Ministry of Education, Culture, Sports, Science and Technology of Japan (23H02869, 23K27560). Other authors declare that they have no known competing financial interests or personal relationships that could have appeared to influence the work reported in this paper.

## Appendix A Supplementary material

.

Supplementary Figure 1. RBE-weighted dose volume histograms and LETd volume histograms for the pectoralis major muscle. (A) Representation of all RBE-weighted dose volume histograms (left) and close-up view of the region with a higher dose (right). (B) Representation of all LETd volume histograms (left) and close-up view of the region with a higher LETd (right). RBE, relative biological effectiveness; LETd, dose-averaged linear energy transfer.

## Footnotes

Appendix A Supplementary material associated with this article can be found in the online version at doi:10.1016/j.ijpt.2025.100746.
