# Supplementary material for: Dose Constraints in Carbon-Ion Radiation Therapy to Minimize the Risk of Pectoral Myositis
Source: Int J Part Ther. 2025 Mar 27;16:100746. doi: 10.1016/j.ijpt.2025.100746 (PMC11999307; doi:10.1016/j.ijpt.2025.100746)
Supplement: Supplementary file 1 — Supplementary material [file mmc1.docx]

**Supplementary table 1. Patient and tumor characteristics and the treatments.**

| Variable | n = 14  (all) | n = 10  (w/o pectoral myositis) | n = 4  (w/ pectoral myositis) | *p*-value |
| --- | --- | --- | --- | --- |
| Median age at enrolment, median (range), years | 57 (50–72) | 56 (50–72) | 59 (55–61) | 0.921 |
| Laterality | Left: 8  Right: 6 | Left: 7  Right: 3 | Left: 1  Right: 3 | 0.348 |
| Tumor size, median (range), mm | 13 (6–19) | 16 (7–31) | 16 (7–31) | 0.816 |
| Tumor area | A: 6  B: 2  C: 5  D: 1 | A: 5  B: 2  C: 3  D: 0 | A: 1  B: 0  C: 2  D: 1 | 0.563 |
| Distance between tumor and skin, median (range), mm | 16 (7–31) | 15 (7–31) | 18 (7–20) | 0.816 |
| C-ion RT RBE-weighted dose, Gy (RBE) | 46 for 3 cases  50 for 11 cases | 46 for 3 cases  50 for 7 cases | 46 for 0 cases  50 for 4 cases | 0.607 |
| PTV, median (range), cc | 8.0 (2.9–14.1) | 10.1 (4.1–14.1) | 6.3 (2.9–8.6) | 0.144 |
| Major pectoralis volume, median (range), cc | 69.6 (26.1–158.6) | 70.0 (39.0–158.6) | 69.6 (26.1–75.4) | 0.254 |

**Abbreviations:**

w/o = without, w/ = with, C-ion RT = carbon-ion radiotherapy, RBE = relative biological effectiveness, PTV = planning target volume.
