# Supplementary figures and images for: Dose Constraints in Carbon-Ion Radiation Therapy to Minimize the Risk of Pectoral Myositis
Source: Int J Part Ther. 2025 Mar 27;16:100746. doi: 10.1016/j.ijpt.2025.100746 (PMC11999307; doi:10.1016/j.ijpt.2025.100746)

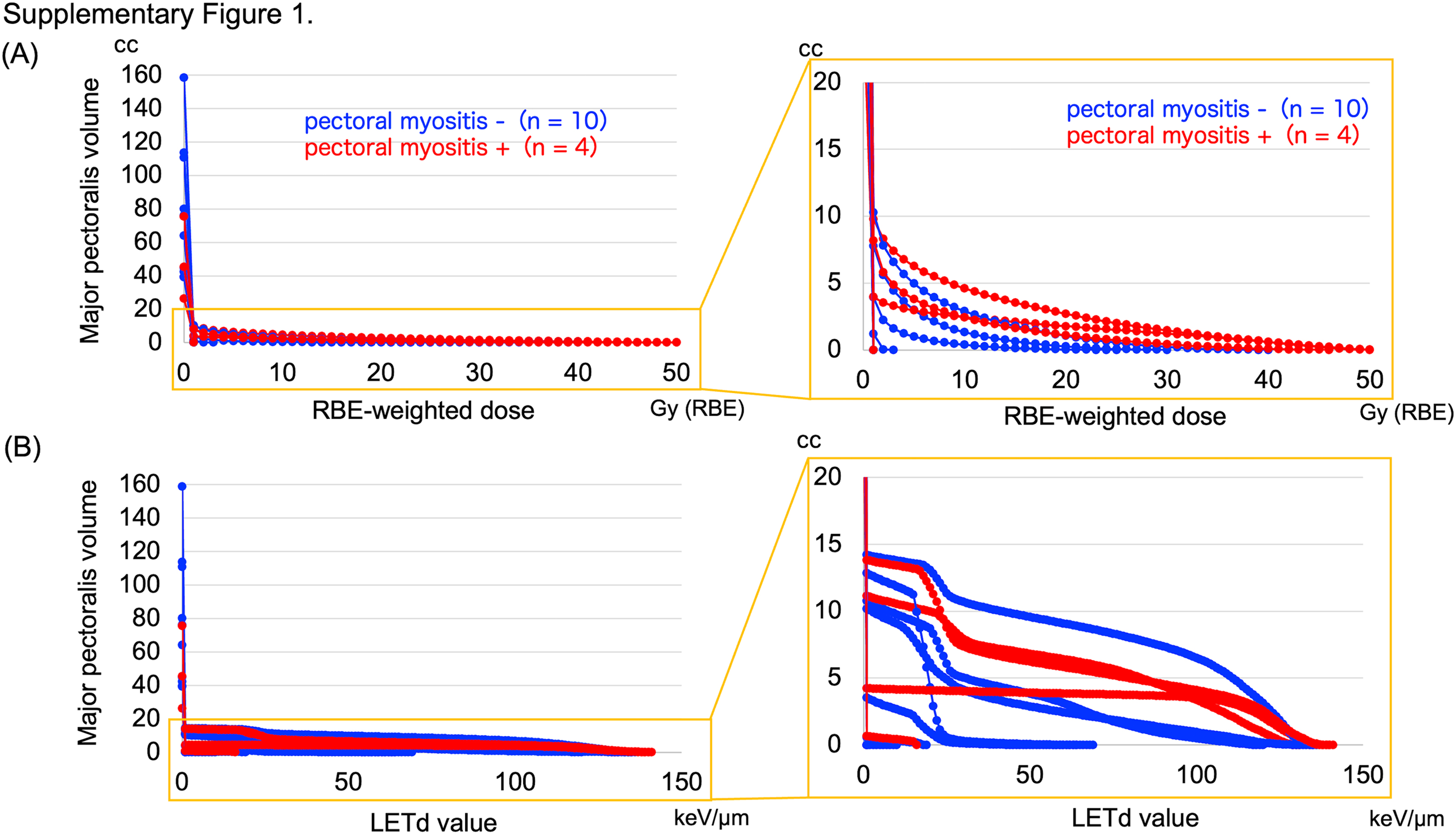

Supplement: Supplementary file 2 — Supplementary Figure 1. RBE-weighted dose volume histograms and LETd volume histograms for the pectoralis major muscle. (A) Representation of all RBE-weighted dose volume histograms (left) and close-up view of the region with a higher dose (right). (B) Representation of all LETd volume histograms (left) and close-up view of the region with a higher LETd (right). RBE, relative biological effectiveness; LETd, dose-averaged linear energy transfer. [file mmc2.jpg]
